# Draft Genome Sequences of the Highly Halotolerant Strain Zygosaccharomyces rouxii ATCC 42981 and the Novel Allodiploid Strain Zygosaccharomyces sapae ATB301^T^ Obtained Using the MinION Platform

**DOI:** 10.1128/MRA.00874-18

**Published:** 2018-08-02

**Authors:** Melissa Bizzarri, Stefano Cassanelli, Leszek P. Pryszcz, Jan Gawor, Robert Gromadka, Lisa Solieri

**Affiliations:** a Department of Life Sciences, University of Modena and Reggio Emilia, Reggio Emilia, Italy; b International Institute of Molecular and Cell Biology, Warsaw, Poland; c Institute of Biochemistry and Biophysics PAS, Warsaw, Poland; University of Arizona

## Abstract

Here, we report draft genome sequences of the halotolerant and allodiploid strains Zygosaccharomyces rouxii ATCC 42981 and Zygosaccharomyces sapae ABT301^T^. Illumina and Oxford Nanopore MinION sequencing revealed genome sizes of 20.9 and 24.7 Mb, respectively. This information will be useful for deciphering the genetics of hybrid adaptation to high salt and sugar concentrations in nonconventional yeasts.

## Abstract (precis)

Here, we report draft genome sequences of the halotolerant and allodiploid strains Zygosaccharomyces rouxii ATCC 42981 and Zygosaccharomyces sapae ABT301^T^. Illumina and Oxford Nanopore MinION sequencing revealed genome sizes of 20.9 and 24.7 Mb, respectively.

## ANNOUNCEMENT

The halotolerant yeasts of the genus Zygosaccharomyces find relevant applications in food spoilage and fermentation (1). They exhibit high diversity in response to high solute concentrations, tendency to hybridization, and ectopic recombination at the mating type loci, leading to ploidy and karyotype variation (2, 3). Zygosaccharomyces rouxii ATCC 42981 is an allodiploid strain isolated from Japanese miso, which grows at NaCl and dextrose concentrations up to 3.0 M and 70% (wt/vol), respectively (4). Zygosaccharomyces sapae represents a novel species, first described in high-sugar traditional balsamic vinegar (TBV), for which ABT301 (= CBS 12607^T^ = MUCL 54092^T^ = UMCC 152^T^) is the type strain (5). ABT301^T^ is a sugar-resistant and slow-growing strain more sensitive to salt than is ATCC 42981. Under standard conditions, ATCC 42981 produces more glycerol than does ABT301^T^ and better retains it in the cell under conditions of salt stress (6). ATCC 42981 is thought to have arisen from hybridization between two divergent parents (3, 5, 7–9), while no evidence about the origin of strain ABT301^T^ is available. Here, we present the draft genome sequences of ATCC 42981 and ABT301^T^.

Single-colony isolates were obtained from the Unimore Microbial Culture Collection (UMCC) of the University of Modena and Reggio Emilia in Italy. ABT301^T^ was isolated from a TBV sample in May to June 2004 (10). DNA was extracted by using the phenol-chloroform-isoamyl alcohol method (11) after cell wall enzymatic lysis with 300 U lyticase (Sigma, St. Louis, MO) and subjected to short-read and long-read sequencing by using the MiSeq (Illumina) and MinION (ONT) platforms. Illumina libraries were prepared with an average insert size of ∼600 bp and sequenced in paired-end mode on a MiSeq instrument using a v3 600-cycle chemistry kit. In total, 2,234,027 and 3,452,971 short paired-end reads were generated for ATCC 42981 and ABT301^T^, respectively.

MinION libraries were prepared from unsheared genomic DNA using a 1D ligation sequencing kit with modifications included in the One-Pot ligation manual (https://doi.org/10.17504/protocols.io.k9acz2e). Genomes were sequenced separately on a MinION MkIb instrument using SQK-LSK108 chemistry and R9.4.1 flow cells. Total numbers of 260,559 and 197,963 long reads were generated for ATCC 42981 and ABT301^T^, respectively. They were basecalled with Albacore v2.1.7, quality trimmed with PoreChop v0.2.1 (https://github.com/rrwick/Porechop) and error corrected with Canu v1.7 (12). Platanus v1.2.4 (13) was used to assemble the initial contigs, which were subsequently scaffolded with corrected MinION reads using DBG2OLC (14). Finally, scaffolds were polished with long reads using Racon v1.2.0 (15) and with short reads using Pilon v1.22 (16) and then reduced using Redundans v.014 (17). All software programs were used at default settings. Genes were annotated by similarity to the closest haploid relative, Z. rouxii CBS 732^T^ (18), using Exonerate v2.2.0 (19). Assembly completeness was assessed by BUSCO v3.0.2 (20).

Comparison with haploid CBS 732^T^ showed that the ATCC 42981 and ABT301^T^ assembled genomes had a 2.14 and 2.53 times larger assembly size and contained a 2.11 and 2.46 times higher number of protein-coding genes, respectively (Table 1). For both genomes we dissected three haplotypes, and one of them was identical to that of CBS 732^T^ (identity cutoff, 0.92). The data suggest a recursive hybridization model (21). The reported assemblies will decipher how hybridization, followed by functional genome stabilization, may offer a rapid adaptation strategy to salt stress environments in yeasts.

**TABLE 1:** Assembly metrics and annotation completeness obtained by using the BUSCO universal fungal genes (fungi_odb9) data set

Feature	Data for strain:		
	CBS 732^T^	ABT301^T^	ATCC 42981
Assembly size (bp)	9,764,635	24,741,993	20,910,059
No. of scaffolds	7	52	33
G+C content (%)	39.13	39.57	39.65
*N*_50_ contig size (bp)	1,496,342	1,409,619	1,393,912
*N*_90_ contig size (bp)	1,114,666	146,869	400,395
No. of gaps	1,269	0	0
Longest scaffold (bp)	1,865,392	1,913,612	1,903,919
No. of genes	4,991	12,300	10,524
No. of BUSCO complete genes (%)	285 (98.28)	282 (97.24)	285 (98.28)
No. of BUSCO duplicated genes (%)	0 (0)	240 (85.11)	264 (92.63)

### Data availability.

The BioProject has been deposited in GenBank under number PRJEB26771. All sequencing reads of Z. rouxii ATCC 42981 and Z. sapae ABT301^T^ have been deposited at EMBL/GenBank under the accession numbers UEMZ01000001 to UEMZ01000033 and UEGL01000001 to UEGL01000052, respectively.
